# Kinesin light chain-4 depletion induces apoptosis of radioresistant cancer cells by mitochondrial dysfunction via calcium ion influx

**DOI:** 10.1038/s41419-018-0549-2

**Published:** 2018-05-02

**Authors:** Jeong-Hwa Baek, Janet Lee, Hong Shik Yun, Chang-Woo Lee, Jie-Young Song, Hong-Duck Um, Jong Kuk Park, In-Chul Park, Jae-Sung Kim, Eun Ho Kim, Sang-Gu Hwang

**Affiliations:** 1 Division of Applied Radiation Bioscience, Korea Institute of Radiological & Medical Sciences, Seoul, 01812 Korea; 2 Department of Molecular Cell Biology, Sungkyunkwan University School of Medicine, Suwon, 440-746 Korea

## Abstract

Kinesins act as molecular microtubule-dependent motor proteins and have various important cellular functions related to cell division, intracellular transport, and membrane trafficking. However, the function of kinesin light chain 4 (KLC4) in cancer, especially radioresistance, has not been previously described. Thus, we investigated KLC4 function in lung cancer cells and radioresistant R-H460 cells by analyzing alterations in radiosensitivity after gene knockdown with siRNA and by evaluating cellular phenotypes and xenograft tumor growth. KLC4 was upregulated in human lung cancer cell lines. Moreover, in paired clinical specimens of lung cancer patients, KLC4 expression was significantly higher in tumor tissues than in paired adjacent normal tissues. Fluorescence-activated cell sorting (FACS) analysis showed that apoptosis rates and cleaved poly (ADP-ribose) polymerase (PARP) and cleaved caspase-3 levels in KLC4-knockdown lung cancer cells were significantly increased compared with those in control cells. Colony formation decreased as the radiation dose increased in KLC4-knockdown lung cancer cells, demonstrating an essential role for KLC4 in radioresistance. Importantly, KLC4 silencing suppressed tumor growth in an in vivo xenograft model, accompanied by increased apoptosis. Finally, KLC4-knockdown cells exhibited impaired mitochondrial respiration, increased mitochondrial reactive oxygen species production, and enhanced mitochondrial calcium uptake, resulting in mitochondrial dysfunction. Thus, KLC4 as a kinesin superfamily-targeted therapy may represent a novel, effective anticancer strategy, particularly for patients showing radioresistance.

## Introduction

Lung cancer is the second most commonly diagnosed cancer and has the highest mortality rate of all types of cancer worldwide^1^. The current best therapies for lung cancer patients achieve anz overall 5-year survival rate of 16 and 6% for non-small cell lung cancer and small cell lung cancer^2^, respectively. Although radiotherapy (RT) is a promising treatment for both early-stage and advanced-stage lung cancer patients, some patients with a high surgical risk experience recurrence and metastatic diseases despite receiving RT treatment^3,4^. A major contributor to poor outcomes is radioresistance; intrinsic (primary) radioresistance involves a subpopulation of clonogenic cells within the tumor^5^, while acquired radioresistance occurs during RT treatment^6^. Furthermore, the mechanisms of cancer radioresistance are affected by several factors that significantly affect radiation efficiency. Thus, identification of radioresistance biomarkers, as well as elucidation of the biological mechanisms underlying radioresistance, is crucial for identifying clinical strategies to improve radioresistant responses to RT.

Human kinesin superfamily members (KIFs) include 14 kinesin families, kinesin-1 to kinesin-14, per the standardized nomenclature developed by the community of kinesin researchers^7^. The members of this family act as molecular microtubule-dependent motor proteins to regulate the distribution of numerous organelles and generate ATP-dependent movement of vesicles, macromolecular complexes, and organelles along microtubules^7–12^. Individual kinesins also play important roles in various cellular functions related to cell division, intracellular transport, and membrane trafficking events, including endocytosis and transcytosis^9–11^. Recently, using proteomics and complementary knockdown analyses to identify radioresistance-related genes, we identified four proteins, namely, plasminogen activator inhibitor type-2, NODAL Modulator 2, Kinesin Light Chain 4 (KLC4), and Procollagen-Lysine, 2-Oxoglutarate 5-Dioxygenase 3.These proteins had not been previously linked to radioresistance^13^. Among all KIFs, the functional form of kinesin-1 comprises a heterotetramer of two kinesin heavy chains (KHCs) and two kinesin light chain (KLCs)^8,12^. Four isoforms of KLC, including KLC1, KLC2, KLC3, and KLC4, have been identified in humans. Kinesin-1 heavy chain comprises an N-terminal globular head (the motor domain) connected via a short, flexible neck linker to the stalk—a long, central alpha-helical coiled coil domain—that ends in a C-terminal tail domain, which is associated with the light-chains^8^. One of these, KLC4 (also known as KNSL8), which comprises 619 amino acids and is encoded on chromosome 14q32.3^9–12^, binds to the heavy chain form and is believed to play a role in a tetrameric microtubule-associated motor protein that produces mechanical force and may be involved in organelle transport, whereby the heavy chains provide the motor activity and the light chains determine the cargo by binding to it^8,12^. However, the function of KLC4 in cancer has not been previously described. In addition, the biological phenotypes related to radiation in cancer therapy have not been identified yet; thus, we investigated the characteristics of KLC4 in cancer.

Mitochondria are reported to be center for ATP synthesis and Ca^2+^ buffering and a source for death signaling molecules, including cytochrome *c*. In addition, loss of mitochondrial potential appears in various cellular destruction pathways, including apoptosis or necroptosis^14–16^. Mitochondrial dysfunction associated with the loss of calcium homeostasis and enhanced cellular oxidative stress are known to play a major role in cell damage^17^. This event is an underlying cause of many human diseases^18^.

In this study, we further investigated the function of KLC4 following small interfering RNA (siRNA) gene knockdown and cellular and xenograft mouse-based analyses in cancer cells. The purpose of the study was to clarify whether KLC4 is a radioresistance biomarker in lung cancer cells and to characterize the underlying mechanisms.

## Results

### KLC4 is involved in radioresistance and tumorigenesis of lung cancer

**Fig. 1:**
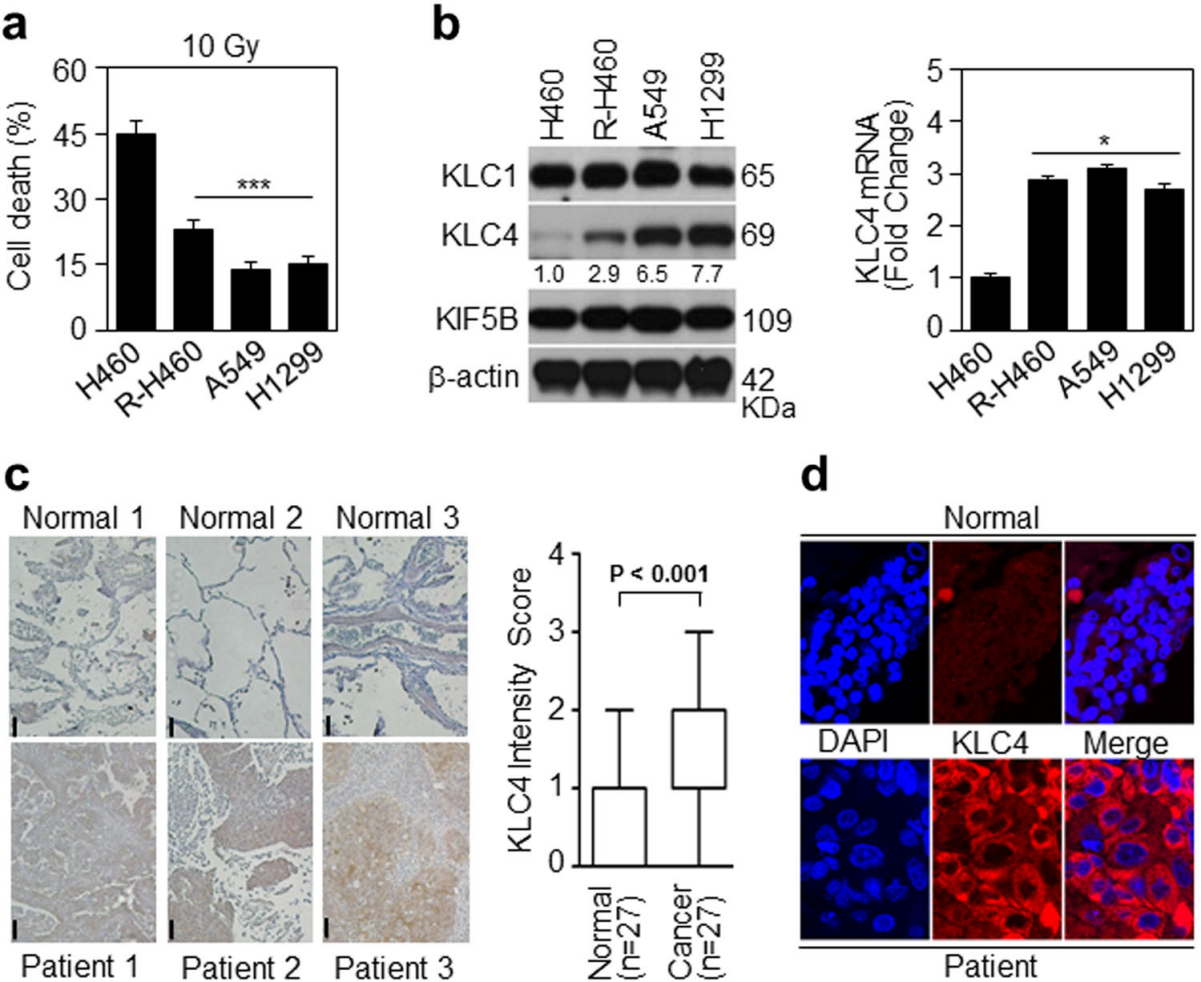
KLC4 protein as a radioresistance and tumorigenesis biomarker. **a** Lung cancer cell lines were treated with 10 Gy radiation for 48 h. Cell death was determined by FACS analysis. **b** In lung cancer cell lines, protein levels of KLC and KHC family members were determined by Western blotting (left) and qRT-PCR (right). **c** Representative microscopic images of lung cancer tissues and their normal tissue counterparts stained with KLC4 antibody (scale bar: 50 μm). Staining intensity was scored as follows: 0, no staining; +1, weak; +2, moderate; and +3, strong. Data are presented as box-and whisker plots. **d** In lung cancer, localization of KLC4 has been visualized by confocal microscopy, and representative images are shown; magnification ×400. All data are shown as the mean ± S.D. of at least three independent experiments. The *P*-values were calculated using unpaired Student’s *t*-test. *P *<* 0.05; **P *<* 0.005; ***P < 0.001

To identify radioresistant lung cancer cells, we assessed the cell death after 10 Gy treatment of H460, R-H460, A549 and H1299 cell lines using FACS analysis (Fig. 1a). In addition, KLC4 mRNA and protein levels in human lung cancer cell lines (H1299, A549, H460 and R-H460) were analyzed by RT-PCR and Western blots. The results showed that the KLC4 level was correlated with radioresistant tendency in the human lung cancer cell lines (Fig. 1b, c). To determine the level of KLC4 in lung cancer patients, we first investigated KLC4 expression in paired clinical lung specimens. The expression of endogenous KLC4 between human lung cancer tissues and paired adjacent non-tumor tissues was analyzed by immunohistochemical staining. As shown in Fig. 1c, among the 27 cases, positive KLC4 expression was observed in tumor tissues compared with the paired adjacent non-tumor tissues (*P* < 0.001, Fig. 1c). The average KLC4 staining score in patient tissues was significantly higher than that in the paired adjacent normal tissues. The results showed that KLC4 was localized to cytosol in the patients samples (Fig. 1d). Altogether, these data indicated that KLC4 was overexpressed in lung cancer, and its overexpression may be associated with radioresistance.

### KLC4 regulates radioresistance in lung cancer cells

**Fig. 2:**
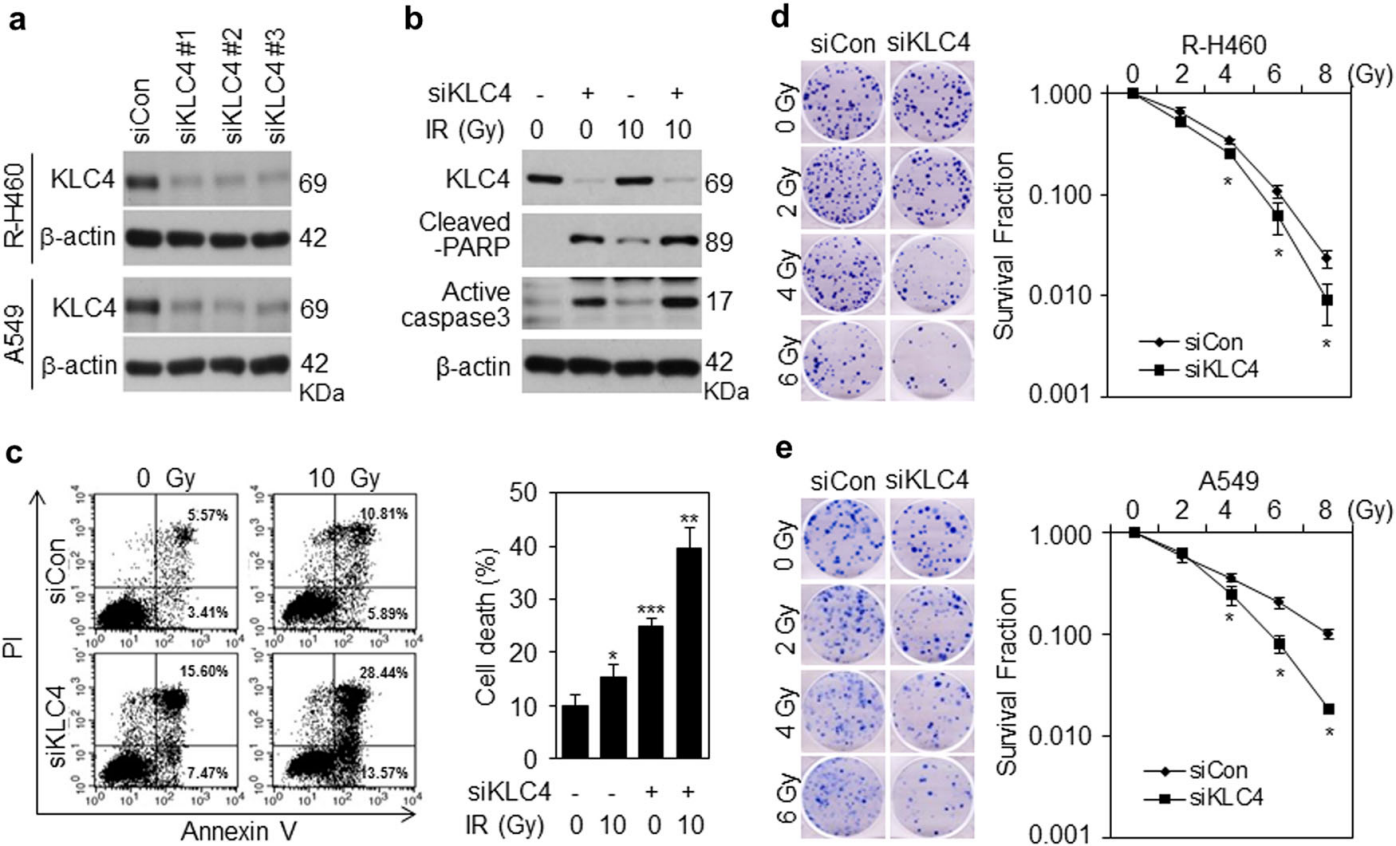
KLC4 depletion with radiation treatment induces apoptosis and anti-proliferative effects in lung cancer cells. **a** Expression of the KLC4 protein by Western blot analysis following transfection with 40 nM control siRNA (siCon) or three different KLC4 siRNAs (siKLC4s) in R-H460 and A549 cells. **b** R-H460 was treated with 10 Gy radiation for 48 h after siKLC4 transfection. Protein levels of cleaved PARP and cleaved caspase3 determined by Western blotting and **c** cell death in R-H460 cells treated as in b determined by Annexin V/PI staining, performed as described in the Materials and Methods. **d**, **e** Surviving fraction of R-H460 and A549 cells treated with a single dose of radiation (0–8 Gy) after transfection with 40 nM control siRNA or KLC4 siRNA, measured 2 weeks after radiation treatment. All data are shown as the mean ± S.D. of at least three independent experiments. The *P*-values were calculated using unpaired Student’s *t*-test. *P *<* 0.05; **P *<* 0.005; ***P *<* 0.001

We examined the ability of specific KLC4 siRNAs to reduce the level of endogenous KLC4 protein in R-H460 and A549 cells with high levels of radioresistance. We transfected R-H460 and A549 cells with three different KLC4-specific siRNAs, and the Western blot analysis results showed that the three KLC4-specific siRNAs had similar levels of efficiency in downregulating KLC4 protein expression (Fig. 2a). Next, to investigate the effect of KLC4 knockdown on apoptosis, we measured the expression of cleaved-PARP and active caspase3, which are apoptotic markers, using Western blot analyses (Fig. 2b). The levels of the two proteins were significantly increased in the R-H460 cells transfected with KLC4 siRNA than in those transfected with control siRNA. Furthermore, FACS analysis was performed to detect the apoptosis rate in R-H460 cells transfected with KLC4 siRNA or control siRNA and administered radiation (Fig. 2c). Forty-eight hours after transfection, the apoptosis rate in R-H460 cells transfected with KLC4 siRNA was significantly increased compared with control siRNA-transfected cells, and this increase was greater in irradiated cells. In addition, the number of colonies formed decreased with an increase in the radiation dose in KLC4 siRNA-treated R-H460 and A549 cells, which indicated a dose-dependent relationship (Fig. 2d, e). Collectively, these results show that KLC4 has an essential role in radioresistance of lung cancer cells.

### Loss of KLC4 promotes radiosensitivity and inhibits tumorigenesis in vivo

**Fig. 3:**
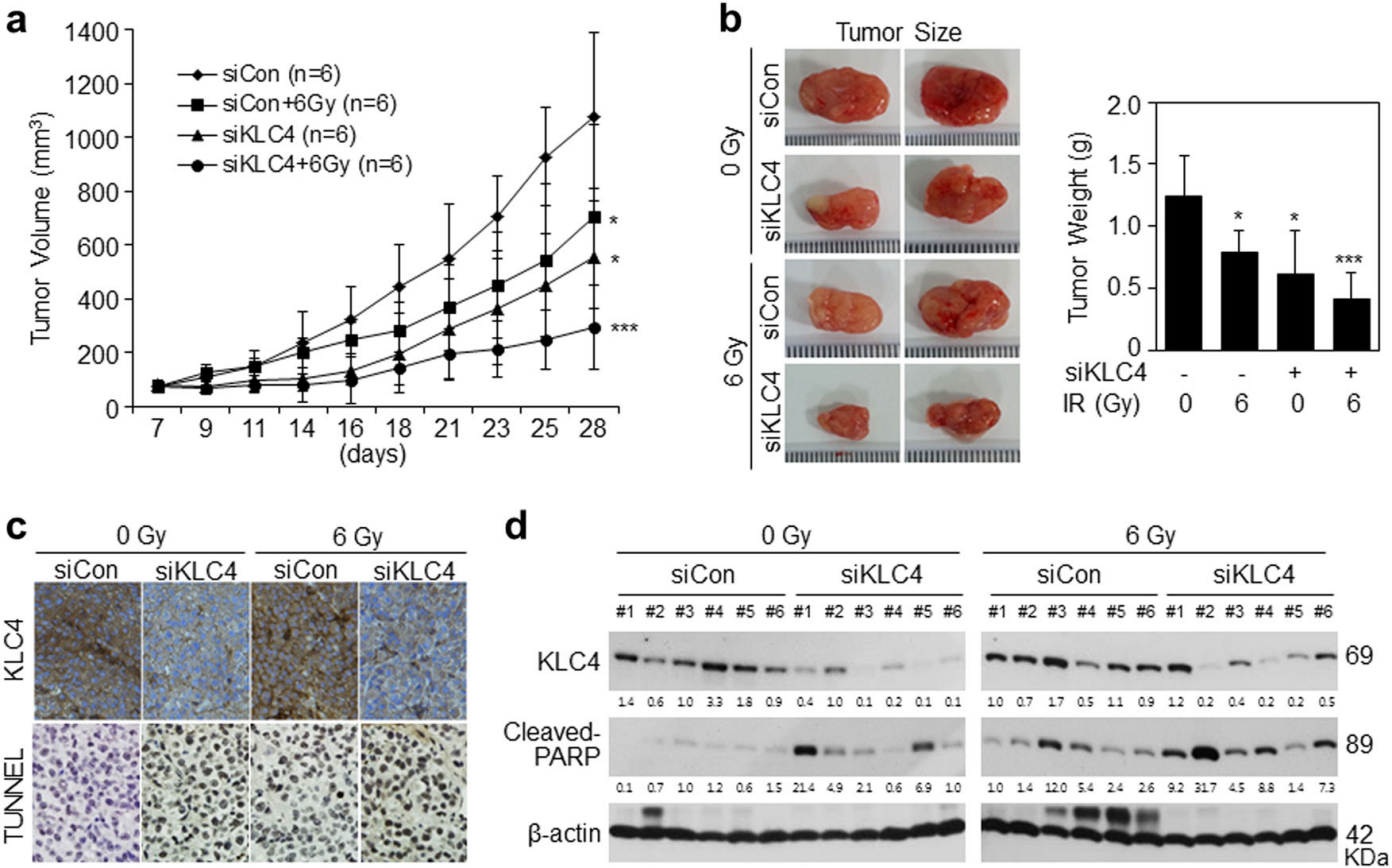
Loss of KLC4 promotes radiosensitivity in vivo. **a** The effect of KLC4 knockdown with irradiation on tumorigenicity by subcutaneous injection of 1 × 10^6^ R-H460 cells. After 7 days, the mice were subjected to tail vein injection every 2 days with control siRNA (siCon) or KLC4 siRNA (siKLC4) (40 μg siRNA/mouse). Ten days after the injection of R-H460 cells, 6 Gy X-ray radiation was delivered to R-H460 xenograft tumors. Tumor volume was calculated at the indicated times using the following formula: Volume = (Length × Width^2^ × 3.14)/6 (*n* = 6). **b** Tumors were excised and weighed at the end of the experiment (28 days). Representative images of tumors and a graph of tumor weight. All data are shown as the mean ± S.D. and the *P*-values were calculated using unpaired Student’s *t*-test. *P *<* 0.05; ***P *<* 0.001. **c** Immunohistochemical staining (top) and TUNEL staining (bottom) of KLC4 xenograft tumor sections after treatment;magnification ×400. **d** Western blot analysis was performed to determine protein levels of KLC4 and cleaved PARP in all samples per group in KLC4 xenograft tumors. All data are shown as the mean ± S.D. of at least three independent experiments. The *P*-values were calculated using unpaired Student’s *t*-test. *P *<* 0.05; **P *<* 0.005; ***P *<* 0.001

After confirming the KLC4 transfection efficiency in vivo, we analyzed the therapeutic potential of KLC4 plus irradiation (IR). KLC4 siRNA was injected into the subcutaneous tumors in nude mice, triggered by R-H460 cells and tumor volume was measured thrice a week. Upon termination of the experiment, the average tumor volume was 1073.5, 705.4, 555.1 and 292.9 mm^3^ for the control siRNA, KLC4 siRNA, IR, and plus IR groups, respectively (Fig. 3a). Differences among the groups were statistically significant. The effects of each treatment on tumor volume and appearance are shown in Fig. 3b. We noticed a significant difference observing the lightest tumor weight in KLC4 siRNA plus IR group (Fig. 3b). The strongest tumor growth inhibitory effect was observed in the KLC4 siRNA plus IR group, which had a tumor inhibition rate of 66.3%. There were no visible signs of toxicity due to KLC4 siRNA and radiation administration in mice, as shown by the lack of differences in body weight and several organs, including the spleen, liver and lung (Supple Fig. 1). In addition, we measured tumor tissue cell apoptosis in vivo by TUNEL assays. The cell apoptosis ratio of the KLC4 siRNA-treated group in vivo was significantly higher than that of the control siRNA group (Fig. 3c). Western blot analysis for the tumors of six mice in each group revealed that the KLC4 siRNA-treated groups had increased protein levels of cleaved PARP in the xenografts, and radiation treatment significantly increased the levels of cleaved PARP despite individual differences (Fig. 3d). These data demonstrated that KLC4 silencing suppressed tumor growth of lung cancer in vivo.

### KLC4 knockdown generates mitochondrial dysfunction in lung cancer cells

**Fig. 4:**
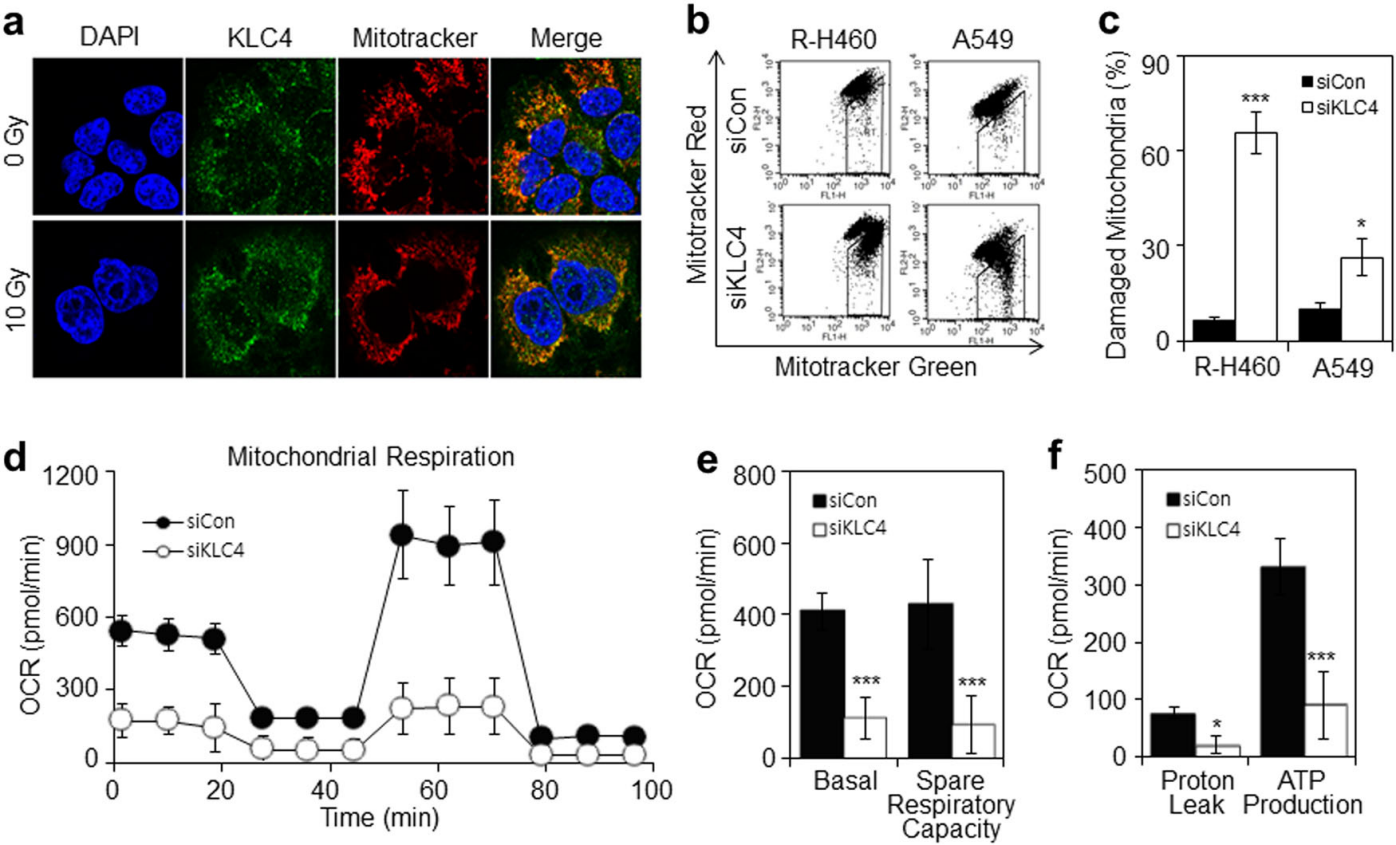
KLC4 knockdown generates mitochondrial dysfunction. **a** R-H460 cells were stained with the KLC4 and MitoTracker Red, a mitochondrial marker, and imaged by confocal microscopy. **b**, **c** KLC4 siRNA-treated cells were analyzed by FACS analysis using MitoTracker Green and Red staining. In **b**, gated cells showed brighter staining with MitoTracker Green, indicating mitochondrial swelling. **d**–**f** R-H460 and A549 cells were treated with KLC4 siRNA, and the OCR was measured using a Seahorse Extracellular Flux Analyzer. Spare respiratory capacity **e** was calculated from the mean of three baseline readings. ATP production rate (**f**) was calculated from the OCR measured in the Seahorse Extracellular Flux Analyzer by the following equat ion: ATP production rate = OCR × 4.6 + PPR x 1. The independent biological experiments were repeated at least three times. Data are represented as the mean ± S.D. from 6 or 7 Seahorse microplate wells. All data are shown as the mean ± S.D. of at least three independent experiments. The *P*-values were calculated using unpaired Student’s *t*-test. *P *<* 0.05; **P *<* 0.005; ***P *<* 0.001

We investigated the intracellular localization of KLC4 in R-H460 cells. Mitochondria were visualized using MitoTracker, and nuclei were stained with 4,6-diamidino-2-phenylindole (DAPI) following treatment. KLC4 was distributed homogeneously in the cytoplasm and mitochondrial regions of untreated cells (Fig. 4a). In addition, to confirm the mitochondrial effects of KLC4 siRNA treatment, we evaluated the extent of KLC4 knockdown-induced mitochondrial damage by FACS analysis using MitoTracker Green and Red staining. The results indicated that KLC4 siRNA-treated R-H460 and A549 cells showed increased mitochondrial damage (Fig. 4b, c). Based on the mitochondrial localization of KLC4 and its effect on cell growth, we investigated changes in mitochondrial function, particularly mitochondrial respiration. The cellular oxygen consumption rate (OCR) was determined using a Sawhorse flux analyzer. Treatment with KLC4 siRNA for 48 h substantially reduced the basal OCR and spare respiratory capacity by 78.4% compared with the control (Fig. 4d, e). The ATP-linked OCR was determined by addition of the ATP synthase inhibitor oligomycin to the cells. As shown in Fig. 4f, ATP-linked respiration was decreased in KLC4 siRNA-treated cells. These results demonstrated that the KLC4 siRNA-treated cells exhibited signs of damage and impaired mitochondrial respiration.

### Loss of KLC4 induces mitochondrial reactive oxygen species (ROS) production and caspase-dependent apoptosis via mitochondrial dysfunction

**Fig. 5:**
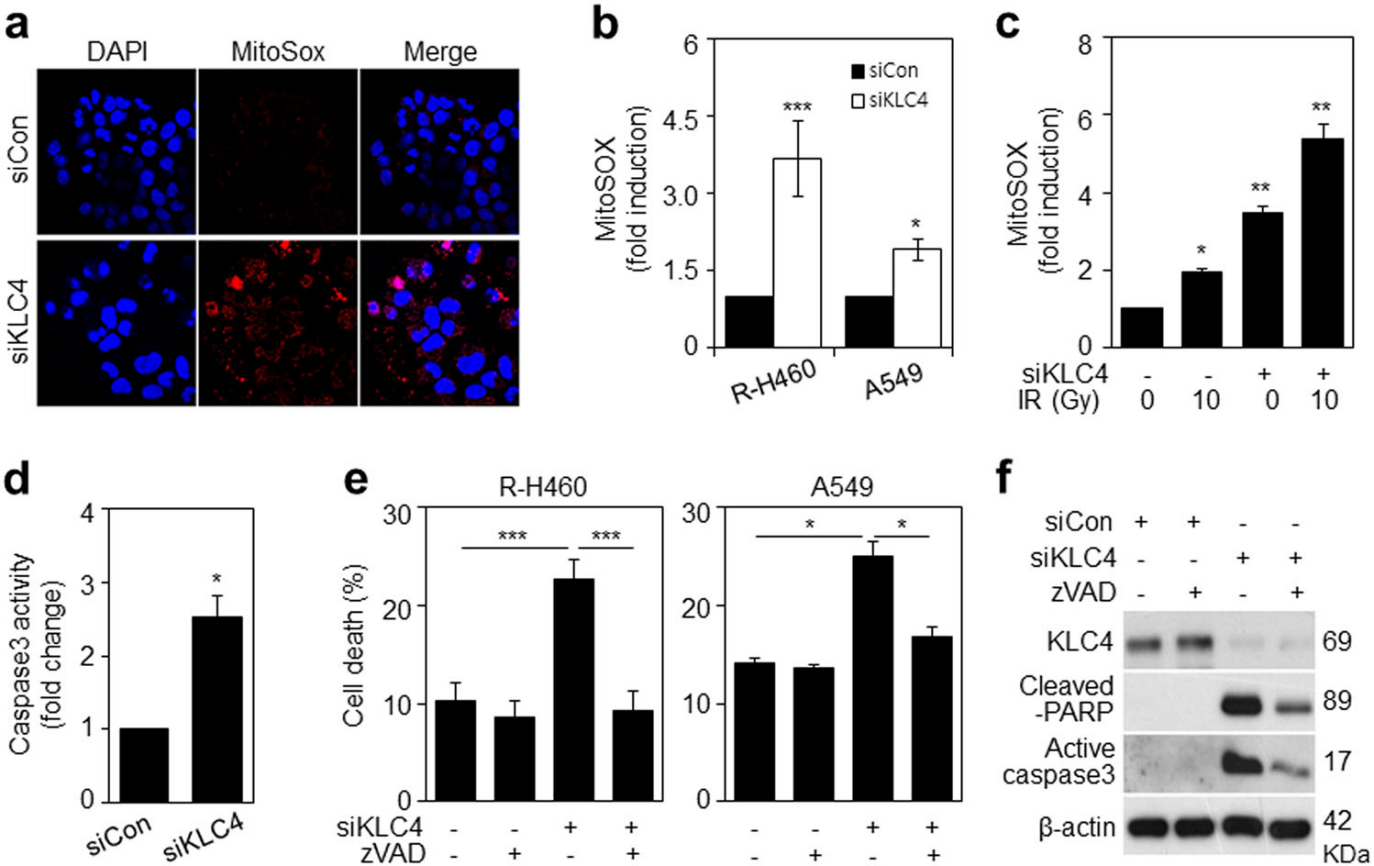
The increased ROS production in mitochondria and caspase-dependent apoptosis in KLC4-depleted cells. **a** KLC4 siRNA-treated R-H460 cells were loaded with the mitochondrion-derived O_2_^–^ indicator MitoSOX Red and imaged by confocal microscopy. Increased MitoSOX fluorescence indicates the production of mitochondrial ROS in cells treated with KLC4 siRNA. **b** KLC4 siRNA-treated cells were analyzed by FACS analysis using MitoSOX Red staining in R-H460 and A549 cells. **c** Mitochondrial ROS analysis of R-H460 cells left untreated or treated with 10 Gy radiation after transfection with KLC4 siRNA, measured 24 h after treatment. **d** Analysis of Caspase activity in R-H460 cells transfected with KLC4 siRNA after 48 h by ELISA. Data were collected using a Multiskan EX (Thermo) at 405 nm. **e** Cell death analysis of R-H460 and A549 cells left untreated or treated with 100 µM Z-VAD-FMK (pan-caspase inhibitor) after transfection with KLC4 siRNA, measured 48 h after treatment. **f** Protein levels of Cleaved PARP and Cleaved caspase3 determined by Western blotting. All data are shown as the mean ± S.D. of at least three independent experiments. The *P*-values were calculated using unpaired Student’s *t*-test. *P *<* 0.05; **P *<* 0.005; ***P *<* 0.001

Next, mitochondrial ROS production was analyzed using MitoSOX, a live cell-permeable and mitochondrial localizing superoxide indicator. KLC4 siRNA-treated R-H460 cells strongly increased the mitochondrial MitoSOX fluorescence (Fig. 5a), indicating that KLC4 knockdown promotes mitochondrial ROS production. Moreover, KLC4 siRNA-treated R-H460 and A549 cells also increased mitochondrial ROS, as shown by FACS analysis (Fig. 5b). This change was enhanced in irradiated cells (Fig. 5c). The activity of caspase-3, which is linked to the apoptosis pathway, was higher in KLC4 siRNA-treated R-H460 cells than in control cells using a caspase assay kit (Fig. 5d). To investigate the detailed mechanism of the KLC4 depletion-induced apoptosis, we added the pan-caspase inhibitor Z-VAD-FMK, and FACS analysis was used to detect the change in cell apoptosis. Notably, the FACS results (Fig. 5e) revealed that the pan-caspase inhibitor Z-VAD-FMK decreased the KLC4 depletion-induced apoptotic cell death in R-H460 and A549 cells. Western blot analysis confirmed that KLC4 depletion induced the activation of PARP and caspase-3 and that Z-VAD-FMK blocked caspase-3 activation (Fig. 5f). Our data suggested that the increased apoptosis by depletion of KLC4 is dependent on caspase activation.

### KLC4 depletion increases mitochondrial dysfunction by mitochondrial calcium uptake

**Fig. 6:**
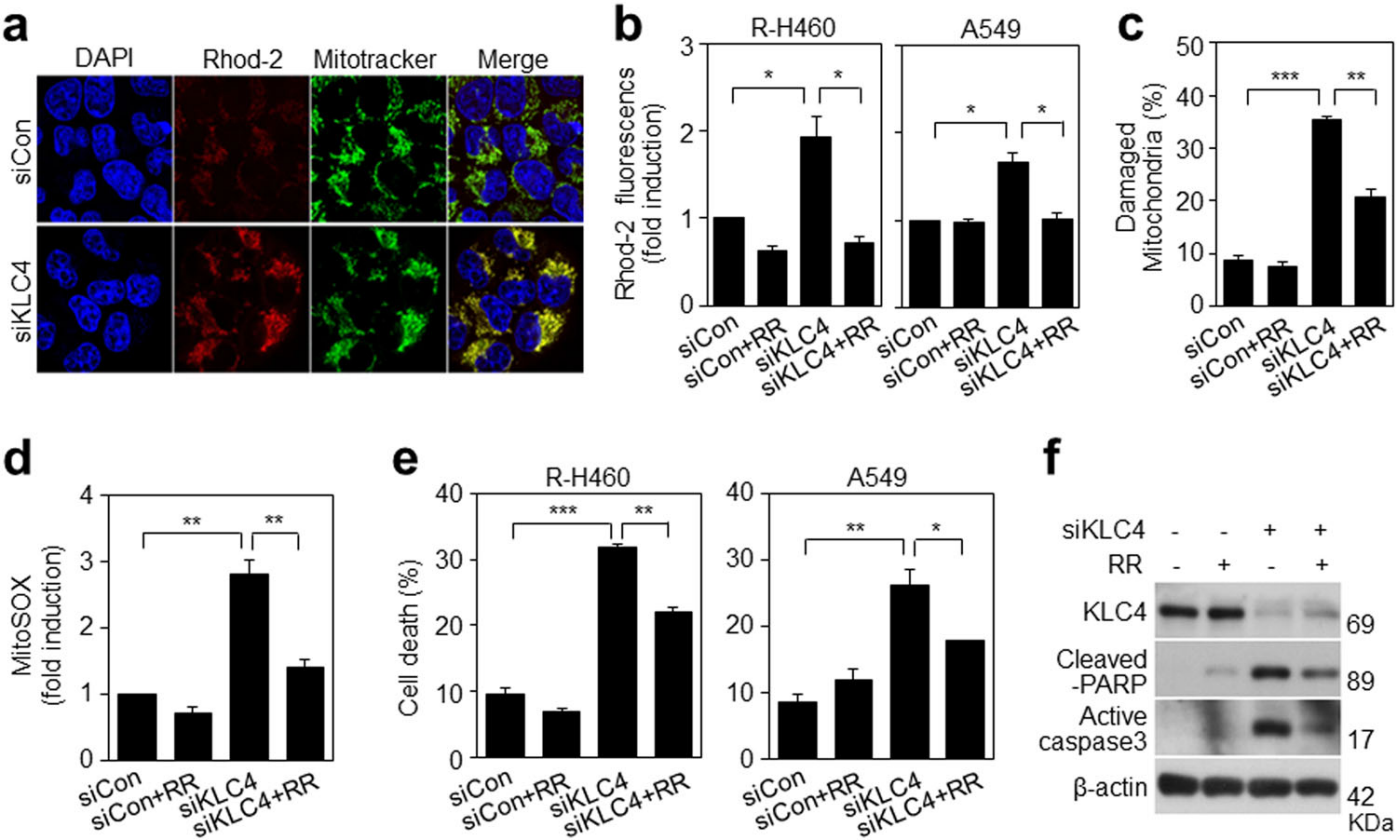
Mitochondrial dysfunction through mitochondrial calcium uptake in KLC4-depleted cells. **a** KLC4 siRNA-treated cells were stained with Rhod-2 and MitoTracker Green, a mitochondrial marker, and imaged by confocal microscopy. **b** Rhod-2 fluorescence determination by FACS analysis after KLC4 siRNA transfection and treatment with ruthenium red (RR) to inhibit mitochondrial calcium uptake in R-H460 and A549 cells. **c** Damaged mitochondria determination by FACS analysis after siKLC4 transfection and RR treatment. **d** Mitochondrial ROS determination by FACS analysis after siKLC4 transfection and RR treatment. **e** The KLC4 siRNA and RR-treated R-H460 and A549 cells were stained with Annexin V and propidium iodide (PI) and then analyzed by FACS analysis. **f** Effects of RR on KLC4 siRNA treated-induced caspase-3 activation and PARP cleavage were analyzed by Western blot analysis. All data are shown as the mean ± S.D. of at least three independent experiments. The *P*-values were calculated using unpaired Student’s t-test. *P *<* 0.05; **P *<* 0.005; ***P *<* 0.001

Calcium uptake into the mitochondrial matrix is critically important to cellular function^19^. We directly measured the effect of KLC4 on mitochondrial calcium uptake using Rhod-2, a selective fluorescent dye for mitochondrial calcium. Colocalization of Rhod-2 staining and MitoTracker Green, the mitochondrial marker, confirmed that the Rhod-2 fluorescence level was increased in KLC4-depleted R-H460 cells (Fig. 6a). And to determine the role of mitochondrial calcium transport, we used an inhibitor of mitochondrial calcium transporters: RR, which inhibits calcium entry via the calcium uniporter. Additionally, KLC4 siRNA-treated cells showed a fold induction in the ratio of Rhod-2 fluorescence in the R-H460 and A549 cell lines using FACS analysis (Fig. 6b). The results showed that KLC4 depletion plus RR recovered the level of Rhod-2 fluorescence, damaged mitochondria and mitochondrial ROS (Fig. 6b–d). Apoptosis rate was also increased in KLC4 siRNA-treated R-H460 and A549 cells and the decrease was prominent in RR-treated cells (Fig. 6e). Furthermore, the expression levels of cleaved PARP and active caspase-3 were correlated with those of FACS analysis in R-H460 cells (Fig. 6f). Thus, KLC4-depleted cells showed altered mitochondrial calcium handling and mitochondrial dysfunction.

### The reproducible KLC4 depletion effect in cervical cancer cell lines

**Fig. 7:**
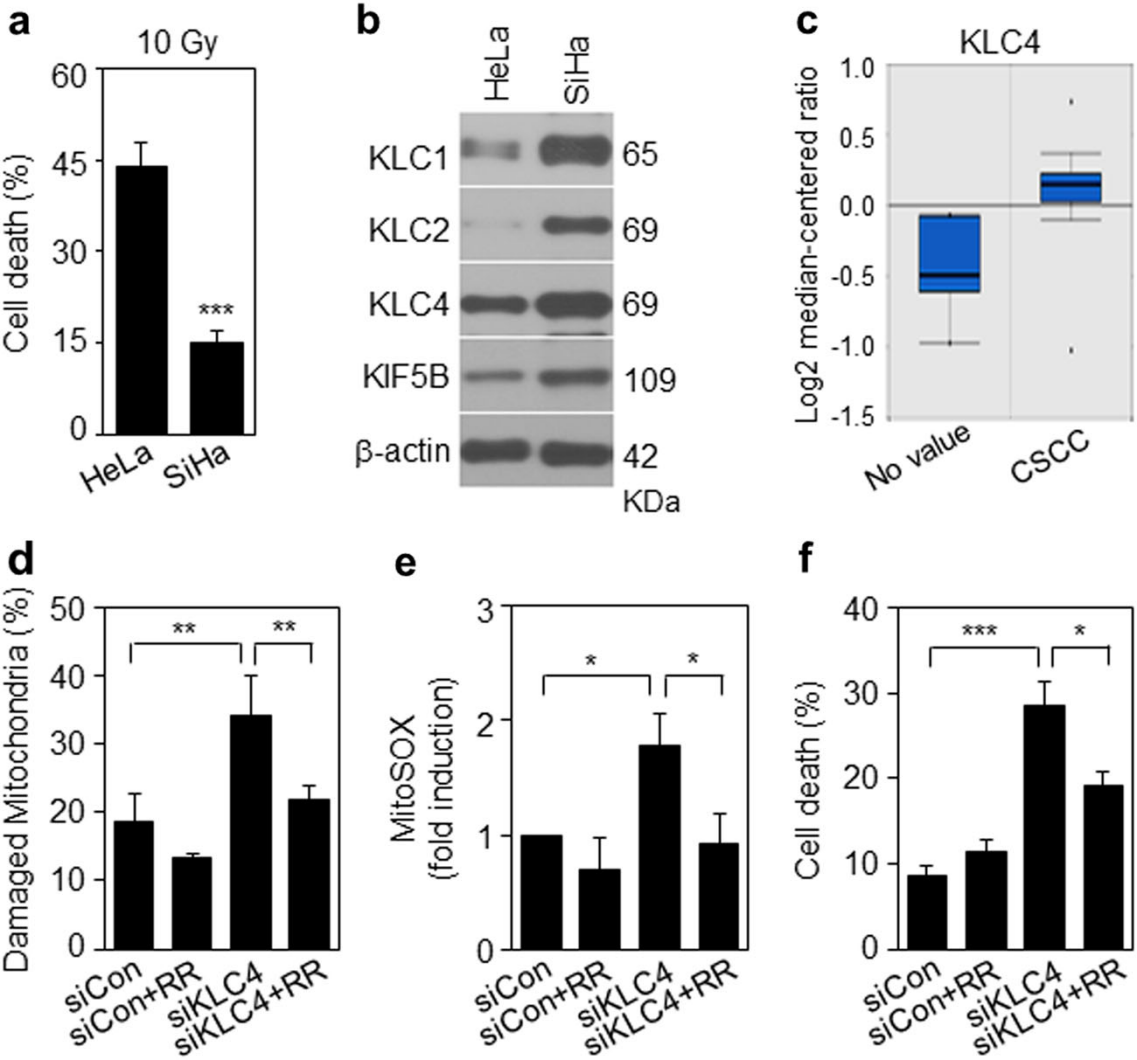
The biological functions of KLC4 in cervical cancer cells. **a** Cervical cancer cell lines were treated with 10 Gy radiation for 48 h. Cell death was determined by FACS analysis. **b** In cervical cancer cells, protein levels of KLC and KHC family members were determined by Western blotting. **c** The available data set in the Oncomine database was queried for KLC4 expression with respect to cervical cancer vs. normal tissues. **d** Damaged mitochondria determination by FACS analysis after siKLC4 plus RR treatment in SiHa cells. **e** MitoSox determination by FACS analysis after siKLC4 plus RR treatment in SiHa cells. **f** The KLC4 siRNA and RR-treated SiHa cells were stained with Annexin V and propidium iodide (PI) and then analyzed by FACS analysis. All data are shown as the mean ± S.D. of at least three independent experiments. The *P*-values were calculated using unpaired Student’s *t*-test. *P *<* 0.05; **P *<* 0.005; ***P *<* 0.001

**Fig. 8:**
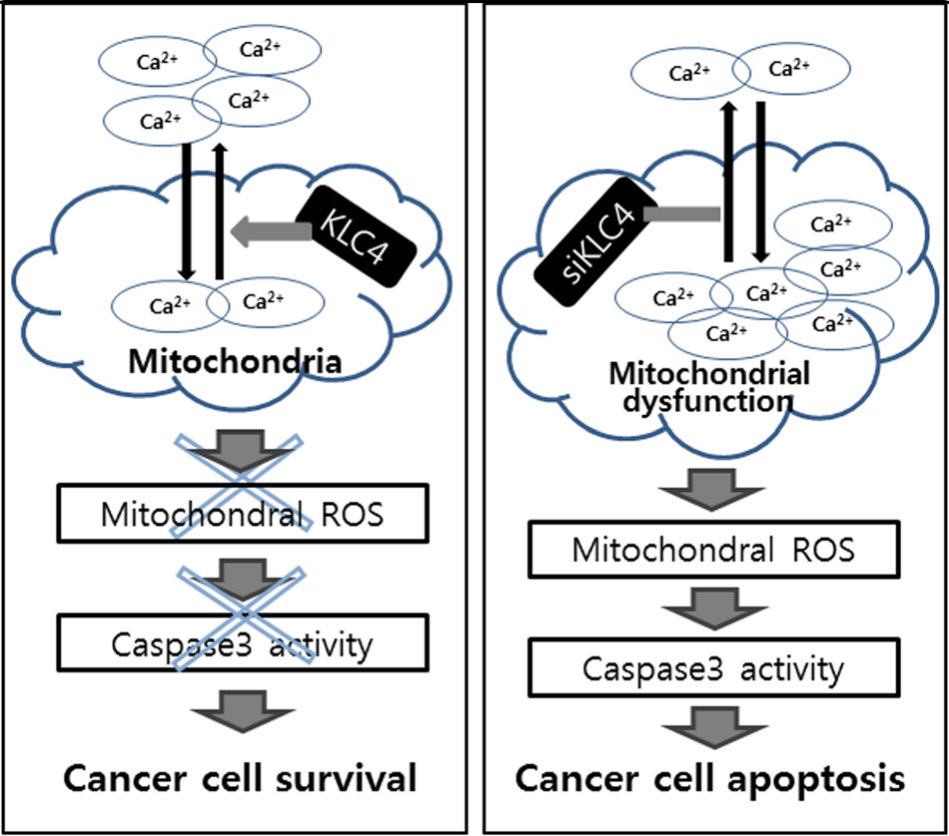
**The proposed scheme for KLC4 as a radioresistance marker**

To investigate whether these phenomena were observed in other cancer types, we used two cervical cancer cell lines, HeLa and SiHa. First, we assessed cell death by FACS analysis after 10 Gy IR treatment. Cell death in these two lines was 44 and 15%, respectively, (Fig. 7a). Consistent with the results of lung cancer lines, the KLC4 protein expression was higher in radioresistant SiHa cells than HeLa cells (Fig. 7b). Furthermore, expression of KCL1, KCL2 and KIF5B except for KLC4 was much higher in SiHa cells than in HeLa cells. Thus, in cervical cancer, since the expression of all KLC family members is increased in SiHa cells, it is necessary to identify the putative function of these genes in relation to radioresistance. Next, we confirmed the correlation between KLC4 expression and cervical cancer severity with the Oncomine database, a human genetic data set analysis tool (Fig. 7c). The results showed that KLC4 depletion plus RR treatment recovered the level of damaged mitochondria, mitochondrial ROS, and cell death (Fig. 7d–f). In summary, the proposed mechanisms for KLC4 as a novel KIF-targeted therapy are shown in Fig. 8.

## Discussion

Although RT is considered an effective treatment protocol for lung cancer patients, the clinical results are not favorable in some cases. This disparity in clinical outcomes is primarily dependent on the radiosensitivity of the type of cancer concerned, and despite the identification of several human radiosensitivity-related genes^20–26^, the detailed mechanisms of radioresistance remain unknown. Thus, radioresistance of lung cancer represents a critical challenge in clinical treatment, and elucidation of the detailed factors that affect lung cancer radioresistance is important for increasing the efficiency of radiation therapy. Therefore, the present study aimed to identify a radioresistance biomarker and the underlying mechanisms that regulate the response of lung cancer to RT.

The aberrant expression of several kinesin proteins in cancer was recently reported to be involved in the malignant phenotype and taxane resistance of solid tumors^27^. Manser et al. showed that lemur tyrosine kinase-2 may play a role in prostate cancer by promoting the binding of Smad2 to KLC2, a light chain of kinesin-1^28–30^. However, until now, the expression pattern of KLC4 in tumor tissue, function of KLC4 in malignant phenotypes, and potential implications of KLC4 expression have not been identified in the context of radioresistance. Thus, we focused on the role of KLC4 in cancers, especially lung cancer, after RT for the first time. With this purpose, we used a siRNA system to determine the radioresistance-inducing effect of the KLC4 protein and to determine whether KLC4 knockdown significantly promotes cell death in radioresistant cell lines.

Our results provide several lines of evidence suggesting that decreased expression of KLC4 plays an important role in cell death-related processes. We showed that siKLC4-treated cells exhibit an increased level of mitochondrial dysfunction through calcium uptake, suggesting that mitochondria participate in apoptosis involving KLC4 in lung cancer cells. We first showed that when highly expressed KLC4 in cancer is knocked down, the major evidence for cell death is related to mitochondrial dysfunction. Key evidence in the present study included the overexpression of KLC4 in several lung cancer cells, particularly in lung cancer patients (Fig. 1), as well as KLC4 depletion-induced apoptosis following radiation treatment in R-H460 cells and the in vivo model (Figs. 2 and 3). Furthermore, the role of KLC4 in promoting cell death was revealed by direct mitochondrial ROS (MitoSOX) stimulation through mitochondrial dysfunction following siKLC4 treatment, indicating the important role of ROS in mitochondrial dysfunction (Fig. 4). Moreover, various evidences suggest that mitochondrial dysfunction plays a key role in oxidative stress^31,32^, and ROS generation further impairs mitochondrial electron transport^33^. Thus, we also showed that KLC4 depletion can lead to a potent influx of extracellular calcium, elevated mitochondrial calcium, increased mitochondrial ROS, and decreased oxygen consumption, all of which preceded the onset of apoptotic cell death; these changes induced a variety of intracellular events, including apoptotic cell death (Figs. 5 and 6). One important factor in mitochondrial dysfunction is accumulation of mitochondrial calcium beyond the transition threshold, which results in opening of the mitochondrial permeability transition pore. This change results in loss of mitochondrial membrane potential, mitochondrial swelling, mitochondrial ROS overproduction, and finally cell death^34–37^. Consistent with these reports, our results showed that calcium uptake with KLC4 depletion plus RR as an inhibitor of the mitochondrial calcium transporters recovered the level of Rhod-2 fluorescence and mitochondrial ROS, but we did not identify how KLC4-depleted cells induce calcium overload. In addition, the ROS production may have induced caspase-dependent expression of KLC4 through activation of the apoptotic pathway in our in vitro and in vivo models. These events included the activation of PARP and caspase-3, which ultimately promote tumor cell apoptosis induced by KLC4 knockdown (Fig. 5). In addition, we confirmed these direct phenomena with cervical cancer cell lines, suggesting the possibility of a widely applicable diagnostic marker in various cancer types (Fig. 7). Thus, our study suggests that KLC4 depletion sensitizes cells to radioresistance through mitochondrial dysfunction via calcium uptake, which is followed by elevated mitochondrial ROS levels and finally by caspase-dependent apoptosis (Fig. 8).

However, important questions remain regarding the involvement of KLC4 in identification of various factors that induce mitochondrial dysfunction. Therefore, further studies required to determine the role in cell death of ROS induction via mitochondrial dysfunction in lung cancer. In addition, we also showed that the increase in mitochondrial ROS by KLC4 depletion is via the p53-independent signal pathway (data not shown). However, our results demonstrated that KLC4 depletion activates p53 in lung cancer cell lines. p53 is involved in the DNA damage response^38,39^. Based on these results, we found that KLC4 localizes to the nucleus and cytosol after radiation treatment, as revealed by the localization of gammaH2AX as a double-strand break marker and KLC4. Hence, further investigation is needed to identify the functions of KLC4 in the DNA damage response.

In conclusion, we identified a new target of acquired radioresistance in lung cancer cell lines. Depletion of KLC4 overcomes the radioresistance of in vitro and in vivo human lung cancer models, and this may significantly enhance the consequence of lung cancer patients, considerably those who exhibit resistance to RT. Successful identification of radioresistant cancer biomarkers can help clinicians to predict RT treatment outcomes and develop an individual therapeutic regimen to increase the benefit of RT for cancer patients. Strategies directed towards the early identification of radioresistant cancer may be more effective in improving the survival of cancer patients than attempting to improve the outcomes of patients once RT failure has already been clinically proven. Elucidation of the biological functions of these radioresistant cancer biomarkers may reveal the mechanisms of radioresistance and contribute to the development of biomarker-guided targeted therapy or combination therapy with the goal of sensitizing cancers to RT, which can also be the basis of new treatment methods. Based on our findings, KLC4 may represent a novel and more effective approach for cancer treatment, especially for those patients who exhibit resistance to RT, and thus, KIF-targeted therapy may be a promising anticancer strategy.

## Materials and Methods

### Cell culture and treatment

Human lung cancer (A549, H460 and H1299) and cervical cancer (SiHa) cell lines were purchased from ATCC (Manassas, VA, USA). Lung cancer cells were cultured in RPMI-1640 medium, and SiHa cells were cultured in Dulbecco's modified Eagle medium (DMEM) supplemented with 10% fetal bovine serum and 1% antibiotic-antimycotic. In this study, we used a radioresistant H460 (R-H460) cell line derived from parental radiosensitive H460 lung cancer cells administered cumulative treatment with 2 Gy radiation twice a week for 20 weeks^13^. Cells were irradiated using a ^137^Cs source (Atomic Energy of Canada, Ltd., Canada) at a dose rate 3.81 Gy/min and treated with 100 μM Z-VAD-FMK (Adipogen life sciences, San Diego, CA, USA) to inhibit caspase activity. Ruthenium red (RR) (Enzo life sciences, Farmingdale, NY, USA) was used to block calcium uptake in mitochondria.

### Annexin V/propidium iodide (PI) staining

Cells seeded at a density of 3 × 10^5^ cells per 60-mm dish were treated with the indicated experimental conditions. For quantification of cell death, cells were trypsinized, washed in phosphate-buffered saline (PBS) and then resuspended in 1× binding buffer. Cells were incubated with annexin V and PI for 15 min and analyzed with a FACScan flow cytometer (BD Biosciences, San Jose, CA, USA).

### Western blot analysis

Western blot analyses were performed as described previously^40^ using the following primary antibodies: KLC4, β-Actin (Sigma, St. Louis, MO, USA), KLC1, KIF5B (GeneTex Inc., San Antonio, TX, USA); cleaved-PARP (Asp214) and active caspase-3 (Cell Signaling Technology Inc., Beverly, MA, USA) as a loading control.

### Quantitative reverse transcription-polymerase chain reaction (qRT-PCR)

Transcripts were quantified by qRT-PCR as described previously^40^ using the following primer pairs: (KLC4), 5′-CAA CAA TTT GGC TGT GCT CT-3′ (sense) and 5′-TTT GCC ACA TCT GGA TGA TT-3′ (antisense); (GAPDH), 5′-CAT CTC TGC CCC CTC TGC TGA-3′ (sense) and 5′-GGA TGA CCT TGC CCA CAG CCT-3′ (antisense).

### Immunohistochemistry

Human tissue microarrays were purchased from SuperBioChips (Cat Number: CC5; Seoul, Korea), and immunohistochemistry was performed using an anti-KLC4 antibody (1:250 dilution; Santa Cruz Biotechnology, Inc.,Santa Cruz, CA, USA) as described previously^41^. Immunostaining was performed using the avidin–biotin–peroxidase method according to the manufacturer’s instructions (Vector Laboratories, Burlingame, CA, USA), and immunofluorescence staining was performed for KLC4. Staining intensity was scored as follows: 0 (no visible staining); 1+ (weak staining); 2+ (moderate staining); 3+ (strong staining).

### Knockdown of proteins by siRNA

The following human-specific siRNAs synthesized as described by the manufacturer (Genolution, Seoul, Korea) were used: siKLC4 #1, 5′-CCG UUC UAU GGA AAA CAU UUU-3′ (sense) and 5′-AAU GUU UUC CAU AGA ACG GUU-3′ (antisense); siKLC4 #2, 5′-GUG UAU CGU GAC CAG AAU AUU-3′ (sense) and 5′- UAU UCU GGU CAC GAU ACA CUU-3′ (antisense); siKLC4 #3, 5′-CCA GAA UAA GUA UAA GGA AUU-3′ (sense) and 5′-UUC CUU AUA CUU AUU CUG GUU-3′ (antisense). A scrambled siRNA that exhibited no significant homology to known gene sequences was used as a negative control. Cells were transfected with 40 nM siRNA in the medium as described previously^40^.

### Clonogenic assay

Cell survival was determined by clonogenic-survival assays as described previously^40^. Briefly, cells were seeded into triplicate 60-mm tissue culture dishes at densities of 2, 4, 8, 16 and 32 × 10^2^ cells/dish and exposed to 0, 2, 4, 6 and 8 Gy, respectively. Cells were exposed once to different doses of radiation. After 14 days, colonies arising from surviving cells were stained with trypan blue solution and counted using a colony counter (Imaging Products, Chantilly, VA, USA).

### Xenograft tumor model

Five-week-old male Balb/c nude mice were purchased from Orient Bio Inc., (Gapyeong, Korea), permitted to acclimate under laboratory conditions for 1 week and fed a nonpurified commercial mouse diet (Superfeed Co., Wonju, Korea) and water ad libitum. Nude mice were allowed to acclimate for 1 week; thereafter, R-H460 lung cancer cells (1 × 10^6^ cells/mouse) were suspended in Matrigel and PBS (1:1). Cells were subcutaneously injected into the nude mice at 6 weeks of age. Each experiment included six mice per group. After injection of cells, the inoculation group was constituted with mice having an average tumor size of 50–100 mm^3^. Mice constituting the negative control or the KLC4 siRNA group were inoculated in vivo with jet-PEI (polyplus) solution. KLC4 siRNA (40 μg/test) and jet-PEI (6.4 μl/test) mixed and inoculated in the tail vein thrice a week. After the first inoculation, a 6 Gy radiation was administered the next day. Tumor dimensions and body weight were measured weekly and tumor growth was evaluated by measurement of the length and the width with electronic calipers, and the tumor volume was calculated using the following formula: Volume = (Length × Width^2^ × 3.14)/6. One month later, the nude mice were sacrificed, and the tumor tissues were excised, weighed and fixed in 4% paraformaldehyde solution for further study. The tumor growth was evaluated by the tumor volume (mean ± S.D.), which was plotted against time. Animal handling was conducted in accordance with the approval of the Animal Care and Use Committee of Korea Institute of Radiological and Medical Sciences (KIRAMS 2015-0070).

### Terminal deoxynucleotidyl transferase-mediated dUTP nick end labeling (TUNEL) staining

Tissue sections of 4-µm thickness were deparaffinized, washed in 100% ethanol for 5 min, rehydrated in decreasing concentration of ethanol, washed twice in PBS, and incubated at room temperature for 30 min with proteinase K solution. Then, 50 µl TUNEL reaction mixture was added, and the slides were incubated for 60 min at 37 °C in a dark humidified atmosphere and washed three times in PBS. Next, 50 µl of Converter-POD was added, and the slides were incubated in a humidified chamber for 30 min at 37 °C and washed three times in PBS. They were stained with DAB, counterstained with hematoxylin and mounted.

### Immunofluorescence confocal microscopy

Immunofluorescence staining for KLC4 (Sigma, St. Louis, MO, USA), and MitoTracker Red staining (Invitrogen, Carlsbad, CA, USA) were performed as described previously^42^. Cell nuclei were identified by staining with DAPI.

### Mitochondrial assays

Cells were treated with siRNA in the experimental conditions and incubated with MitoSOX (10 min), Rhod-2 AM (30 min), MitoTracker Green and Deep Red (30 min; Invitrogen, Carlsbad, CA, USA). Data were acquired on a FACScan flow cytometer and with immunofluorescence staining.

### Measurement of OCR

Cells were treated with siRNA in the experimental conditions and seeded into 24-well plates (1.5 × 10^4^ cells/well). Cells were washed with Seahorse assay media (300 mg/L l-glutamine, 2000 mg/L d-glucose) and incubated with no CO_2_ 37 °C. OCR was measured according to the manufacturer’s recommendations, followed by oligomycin (1 μM), FCCP (1 μM), and antimycin A (0.5 μM) addition.

### Caspase activity assay

Caspase activities were measured using caspase family activity assay kits (Abcam, Cambridge, UK) according to the manufacturer’s recommendations. Data were collected using a Multiskan EX (Thermo Scientific, Karlsruhe, Germany) at 405 nm.

### Oncomine data mining

Oncomine database (www.oncomine.org) was used for data analysis and visualization as described previously^43^. KCL4 expression was compared between cervical cancer and normal tissue extracts.

### Statistical analyses

Cell culture experiments were repeated at least thrice. All data are expressed as the mean ± standard deviation. Statistical differences between groups were assessed by Student’s *t*-test, and a *P*-value < 0.05 was considered significant.

## Electronic supplementary material

SUPPLEMENTARY FIGURE LEGEND

SUPPLEMENTARY FIG1
