# Supplementary material for: Kinesin light chain-4 depletion induces apoptosis of radioresistant cancer cells by mitochondrial dysfunction via calcium ion influx
Source: Cell Death Dis. 2018 May 2;9(5):496. doi: 10.1038/s41419-018-0549-2 (PMC5931584; doi:10.1038/s41419-018-0549-2)
Supplement: Supplementary file 1 — SUPPLEMENTARY FIGURE LEGEND [file 41419_2018_549_MOESM1_ESM.docx]

**Supplementary** **Figure Legends**

**Supplementary Fig1.** (a) Effects of siRNA and radiation on body weights. Data shown are the mean of mice in each group. (b, c, d) Spleen, liver and lung of mice were excised and weighed at the end of the experiment (28 days) for control siRNA, KLC4 siRNA, IR, and plus IR groups.

All data are shown as the mean ± S.D and the *P*-values were calculated using unpaired Student’s t-test. **P* < 0.05.
