# Supplementary figures and images for: Kinesin light chain-4 depletion induces apoptosis of radioresistant cancer cells by mitochondrial dysfunction via calcium ion influx
Source: Cell Death Dis. 2018 May 2;9(5):496. doi: 10.1038/s41419-018-0549-2 (PMC5931584; doi:10.1038/s41419-018-0549-2)

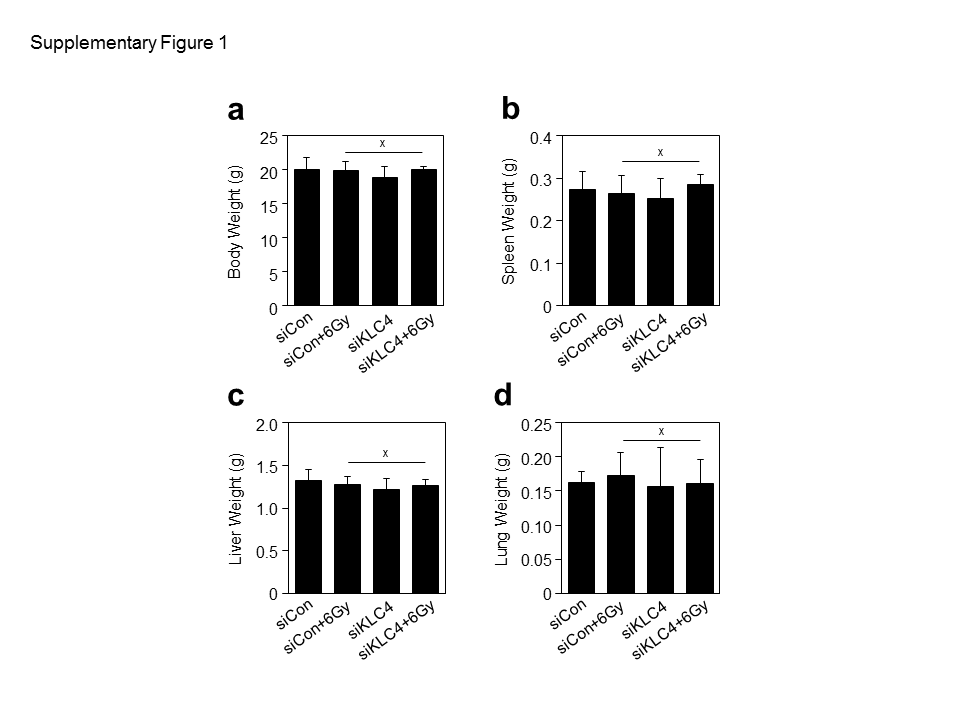

Supplement: Supplementary file 2 — SUPPLEMENTARY FIG1 [file 41419_2018_549_MOESM2_ESM.tif]
